# Mini review: Enzyme-based DNA synthesis and selective retrieval for data storage

**DOI:** 10.1016/j.csbj.2021.04.057

**Published:** 2021-04-25

**Authors:** Eojin Yoo, Donghui Choe, Jongoh Shin, Suhyung Cho, Byung-Kwan Cho

**Affiliations:** a Department of Biological Sciences, Korea Advanced Institute of Science and Technology, Daejeon 34141, Republic of Korea; b Innovative Biomaterials Research Center, KI for the BioCentury, Korea Advanced Institute of Science and Technology, Daejeon 34141, Republic of Korea

**Keywords:** Enzymatic DNA synthesis, DNA data storage, Single-stranded DNA, Terminal deoxynucleotidyl transferase, Synthetic Biology

## Abstract

The market for using and storing digital data is growing, with DNA synthesis emerging as an efficient way to store massive amounts of data. Storing information in DNA mainly consists of two steps: data writing and reading. The writing step requires encoding data in DNA, building one nucleotide at a time as a form of single-stranded DNA (ssDNA). Once the data needs to be read, the target DNA is selectively retrieved and sequenced, which will also be in the form of an ssDNA. Recently, enzyme-based DNA synthesis is emerging as a new method to be a breakthrough on behalf of decades-old chemical synthesis. A few enzymatic methods have been presented for data memory, including the use of terminal deoxynucleotidyl transferase. Besides, enzyme-based amplification or denaturation of the target strand into ssDNA provides selective access to the desired dataset. In this review, we summarize diverse enzymatic methods for either synthesizing ssDNA or retrieving the data-containing DNA.

## 1 Introduction

In synthetic biology, the two major topics being highlighted are DNA synthesis and its applications in the data storage systems. We are experiencing a ‘data explosion’, which refers to a state when massive amount of data is rapidly generated and stored in a computer [1]. Thus, we need to develop an efficient method of storing digital data, and DNA is sought to be the most attractive candidate for replacing the current data storage mediums.

The idea of ‘genetic memory’ first came up in the 1960 s by Nobert Wiener. Since then, the growth of storing data in DNA is accelerated as both the storage capacity and synthetic scale have been enlarged by Church and Goldman, storing 659 KB and 739 KB of data in DNA molecules, respectively [2], [3]. The largest size of data reported is presented by Strauss and Ceze, encoding 200 MB of data in a DNA library [4]. With such advances in synthetic biology technology, a number of creative and practical techniques for storing DNA data have been proposed (Table 1) [4], [5], [6], [7], [8].

**Table 1:** Comprehensive lists of DNA data storage system.

**Total data**	**Code**	**Contents**	**Synthesis method**	**Length (nt)**	**Bits per nucleotide**	**Random access**	**Ref.**
650 kB	1 bit to 1 base	53,426 English text 11 JPG images 5.27-megabit computer code (all as HTML encoded draft)	Phosphoramidite (silicon-based)	115	0.6	No	[2]
739 kB	8 bits to 5–6 bases (ternary code, rotating encoding)	Text file of Shakespeare’s sonnets PDF file of scientific paper JPEG file of the European Bioinformatics Institute MP3 file from Martin Luther King’s 1963 ‘I have a dream’ speech a Huffman code	Phosphoramidite (microarray-based)	117	0.19	No	[3]
83 kB	Indexing and Reed–Solomon coding	Text from the Swiss Federal Charter from 1291 and the English translation of the Method of Archimedes	Phosphoramidite (microarray-based)	158	0.86	No	[6]
151 kB	8 bits to 5–6 bases (ternary code, rotating encoding)	Three JPG files	Phosphoramidite (column-based)	120	0.57	Yes	[58]
2 MB	DNA fountain encoding	Text file of amazon gift card Kolilbri operating system SVG file of pioneer PDF file of shannon's manuscript Video file of the arrival of a train Zipbomb malware	Phosphoramidite (silicon-based)	152	1.18	No	[105]
22 MB	6 bis to 3 bases, 2 bits to 2 bases	MPEG compressed movie sequence	Phosphoramidite (microarray-based)	230	0.89	No	[106]
200.2 MB	Indexing and Reed–Solomon coding	35 distinct files, including high-definition video, images, audio, and text. These included the “Universal Declaration of Human Rights” in over 100 languages	Phosphoramidite (silicon-based)	150–200	0.81	Yes	[4]
33 kB				150			
8.5 MB	2 bits to 1 base	Short input message in ASCI code	Phosphoramidite (silicon-based)	194	1.94	No	[107]
854B	One character to DNA codon	Text file	Phosphoramidite (column-based)	85	1.78	No	[108]
3 kB	14 bits to 8 bases	Two JPEG images, The Citizen Kane poster and Smiley Face	Phosphoramidite (column-based)	880–1,000 (assembly of 100nt)	1.71	Yes	[109]
18B	2 bits to 1 base (ternary code,rotating encoding)	Text message	Enzyme-based (TdT, Apyrase)	150–200	1.5^a^	No	[42]
110B	2 bits to 1 base (ternary code,rotating encoding)	Sheet music	Enzyme-based (TdT, DMNP-EDTA)	50–100	9.2^a^	Yes	[5]

a Bits per transition.

**Fig. 1:**
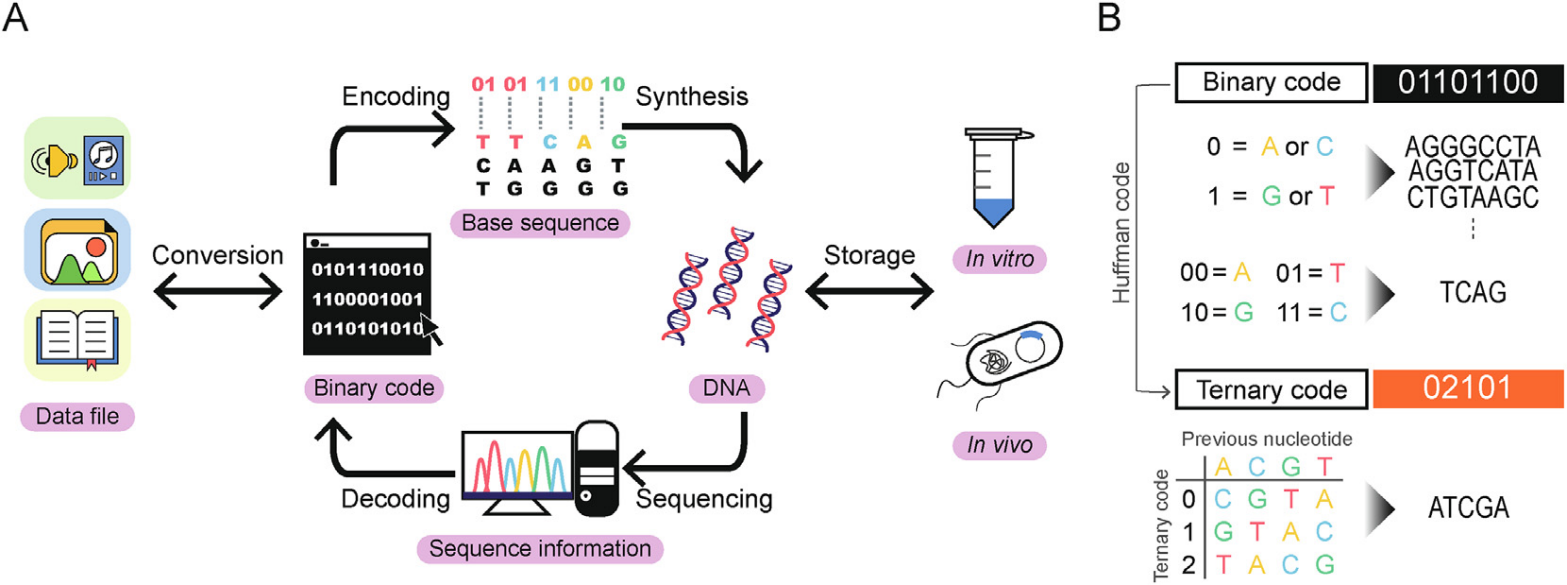
**Overview of DNA data storage system**. (A) The schematic mechanism of the whole process. To begin, data files are converted into binary code and sequentially encrypted in DNA sequence, using the coding scheme (e.g. Huffman code). Designed sequences are synthesized into DNA with enzymatic methods. After long-term storage *in vitro* or *in vivo*, data-containing DNA is sequenced and decoded for complete recovery of the file. (B) Conversion of digital data into DNA sequence. Binary code can be assigned to represent either two bases (upper) or one base (lower). Ternary code, generated by Huffman coding, is used for rotating encoding.

The current DNA data storage process mainly consists of writing, storing, and reading steps (Fig. 1). To begin the process, data files are converted to binary data consisting of 0 s and 1 s and stored in the form of nucleotides. Based on the designed sequence, the ssDNA is synthesized by linking nucleotides in order. This user-defined ssDNA is stored *in vitro* or used to make dsDNA for long-term storage. In order to extract data from the stored DNA, the target sequence is retrieved by selective amplification through PCR and sequenced using next-generation sequencing (NGS) methods. After error detection, the reads are decoded to the original data code for determining successful recovery.

There have been many reviews that discussed the data storage procedures. However, as recent studies are transitioning to develop novel methods using enzymes, we would like to shed light on enzymatic methods which could be potentially used in data storage. Although most of the methods described here lack precedent usage in data storage, we strongly believe they could be applied to future enzymatic DNA data storage. In this review, under the three steps of the data storage system shown in Fig. 1, we have focused on the data writing and reading steps. With the emphasis on using enzyme-based techniques, we would like to present how data-encoding DNA is synthesized and selectively retrieved in the form of single-stranded DNA (ssDNA).

## 2 Writing: Encoding data in DNA

DNA synthesis for data storage has long been relying on the phosphoramidite method, which is a four-step cyclic reaction involving the addition of the desired nucleotide to a growing oligonucleotide chain immobilized on solid support [9], [10]. Based on the automation and improvement of the phosphoramidite method, an oligonucleotide synthesizer can synthesize up to 200 nucleotides (nt) with a 99.3% yield per synthesis cycle [11], [12]. However, this method has unresolved limitations for several reasons. First, the longer the oligonucleotide sequence, the lower the final product yield becomes. Theoretically, even 99% of the nucleotide incorporation efficiency per reaction cycle will yield 36.7 and 13.4% of the full-length product after 100 and 200 cycles (0.99^100^ and 0.99^200^), respectively [13], [14]. The final yield of oligonucleotide will be even lower than the theoretical yield, depending on the purification methods, such as MOPC, HPLC, and PAGE. Thus, current chemical DNA synthesis is limited to 200 nt as its upper limit, which may hinder the encoding of vast amounts of data in a sequence. Second, chemical synthesis requires the use of anhydrous solvents, which produce toxic wastes [15], [16]. Additionally, the use of either trichloroacetic acid or dichloroacetic acid causes depurination of adenosine. Since depurination forms an abasic site on the sequences, it damages DNA under the deprotection step by cleaving or truncating the product which is critical for generating error-free data [17], [18]. Therefore, as an alternative to chemical synthesis, a bio-based DNA synthesis method is highlighted.

Enzyme-based data storage is still in its infancy, but it is expected to involve lesser costs and time for the synthesis of data-containing DNA, compared to the chemical synthesis of DNA. The concept of enzymatic DNA synthesis arises from the discovery of DNA polymerase [19], [20]. However, to have a user-defined DNA sequence like in the chemical method, we need enzymes capable of extending the 3′ end of the ssDNA in a template-independent manner, such as polynucleotide phosphorylase (PNPase), T4 RNA ligase, and TdT.

### 2.1 Polynucleotide phosphorylase (PNPase)

**Fig. 2:**
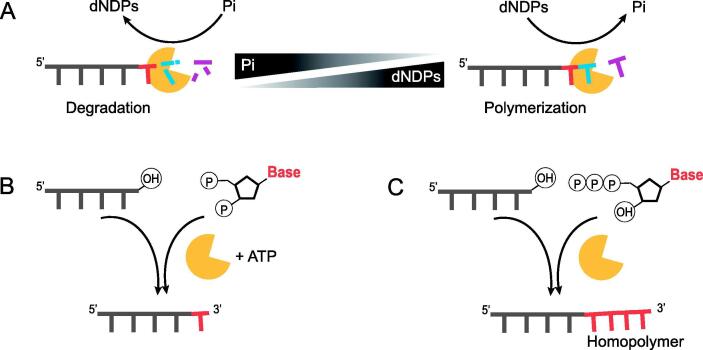
**Schematic illustration of enzymatic ssDNA synthesis**. (A) PNPase; Mechanism of PNPase depends on dNDPs/Pi concentration. (B) T4Rnl; T4Rnl ligases 3′ hydroxyl group on the acceptor and 5′ phosphate of the incoming nucleotide, using ATP. 2′-deoxyribonucleoside 3′,5′-bisphosphate allows sequence-specific addition of one nucleotide per a cycle. (C) TdT; TdT is capable of generating homopolymeric sequence.

PNPase was originally known for its processive RNA degradation activity [21], [22]. However, it has been demonstrated that PNPase is responsible for the polymerization of ribonucleoside diphosphates when Mg^2+^ ions are present. PNPase uses nucleoside diphosphates (NDPs) to perform template-independent synthesis of RNA, releasing orthophosphate (Pi) as its side product (Fig. 2**A**) [22]. To synthesize a defined RNA sequence using PNPase, a method was proposed to use modified nucleotides with a blocker attached to it which prevents the addition of additional enzymes in the sequence, allowing only one at a time [23]. The four requirements of the blocking group they proposed are as follows: i) It should be chemically stable under enzymatic reactions. ii) Chemical deblocking must be possible and not affect the structure of the growing polymer. iii) The blocking group must be small enough to be used as a substrate by the enzyme. iv) The size and configuration of the synthesized polymer with a blocking group on its 3′ end should be durable enough to block further extension. Following these four entries, a stepwise synthesis of ssDNA using 2′(3′)-O-(α-methoxyethyl) nucleoside 5′-diphosphates or 2′(3′)-O-isovaleryl derivatives of NDPs was suggested [24], [25], [26], [27], [28]. To enhance the specificity towards dADP and balance the phosphorlysis and polymerization activity, Mn^2+^ concentration and the ionic concentration were optimized, respectively, which succeeded in adding nine dNDPs to chemically synthesized 4-mer nucleotides [29]. Although recent studies have shown that PNPase has the potential to be used for ssDNA synthesis, PNPase had a critical drawback that it also catalyzes the reverse reaction. The direction of reversible PNPase reaction highly depends on the relative concentration of orthophosphate and dNDPs [30].

### 2.2 T4 RNA ligase (T4Rnl)

T4Rnl forms a phosphodiester bond between the 5′ phosphate and the 3′ hydroxyl group of the oligonucleotides in an ATP-dependent manner (Fig. 2**B**) [31], [32]. As it connects the 5′ and 3′ ends of the chains which are present as the reactants in series, DNA synthesis with T4Rnl is limited to the formation of homopolymeric molecules [33]. Therefore, in order to synthesize the desired sequence, a method was developed using 2′-deoxyribonucleoside 3′,5′-bisphosphate to the 3′ hydroxyl group of the oligonucleotide and block any further extension by 3′-phosphate [34]. When synthesizing deoxyribonucleoside bisphosphate to the oligodeoxyribonucleotide, excessive donor concentrations and low ATP levels helped yielding more than 85% per cycle. However, the time required for the reaction was 5 to 21 days, which is inefficient for synthesizing 10 or more nucleotides [35]. The reaction time was shortened through solid-state synthesis, resulting in a reduction in the reaction time to less than 144 h [36]. In addition, the synthesis efficiency varies widely depending on the type of reaction substrates [36], [37], and the presence of a 3′ unblocked donor is likely to cause self-ligation and circulation. Therefore, even if T4Rnl synthesizes ssDNA for a certain length, it needs to be further optimized for fast and efficient long-chain DNA synthesis.

### 2.3 Terminal deoxynucleotidyl transferase (TdT)

TdT is an enzyme belonging to the DNA polymerase X family. Unlike other polymerases, it has a unique property that enables template-independent DNA synthesis [38], [39]. There are two main reasons why TdT has template-independent properties. From a mechanistic viewpoint, the TdT reaction can occur between the 3′ OH and triphosphate groups of the incoming nucleotide, without the need to specifically bind a complementary base on the template strand. Another reason arises from a notable structural variation of the existence of a ‘lariat-like’ loop [40], [41]. The crystal structure of TdT demonstrates that this lariat-like loop, which consists of 16 amino acids, acts as a physical obstacle, generates steric hindrance, and prevents the accommodation of dsDNA. However, one limitation of using TdT is the formation of a homopolymer tail (Fig. 2**C**). Since TdT actively adds nucleotides to the terminal sequence, as long as the reaction conditions meets, there is no way to block further extension of nucleotides. Thus, the unique approaches of using TdT have been presented by either controlling homopolymer production or adding a single modified nucleotide.

#### 2.3.1 Competitive synthesis

Based on the enzyme kinetics of wild-type TdT, it rapidly synthesizes DNA with natural dNTPs. A competitive addition of nucleotides was demonstrated to utilize the formation of homopolymers in data storage [5], [42]. Either enzymes or ion-caging molecules was used to block TdT activity at a level where they could control the number of nucleotides added. Instead of having a perfect sequence for representing each bit, the method aimed to record the rotational ternary code between the base transition sites. In this strategy, TdT competes with apyrase, an ecto-nucleoside triphosphate diphosphohydrolases for access to the nucleotides available. As a nucleotide converter, apyrase intensely degrades dNTPs to deoxynucleoside diphosphates (dNDPs) or dNMPs (monophosphate). Since TdT has a higher preference for enzyme activity with dNTPs than dNDPs [43], the presence of apyrase in the reaction prevents TdT from incorporating dNTPs. Based on the nature of TdT, it was attempted to control the activity of TdT by converting dNTPs to dNDPs [42]. By this method, TdT can only use the nucleotides before being degraded by apyrase, and the number of nucleotides added is three to five bases on average [44].

Similar to the apyrase method, 1-(4,5-dimethoxy-2-nitrophenyl)-1,2-diaminoethane-N,N,N',N'-tetraacetic acid (DMNP-EDTA), a chelator, competes intensively with TdT for catalytic ions in the reaction [5]. TdT uses catalytic ions for the nucleophilic attack, as cobalt ions are known to contribute most to enzyme activity by increasing the rate of base incorporation at the catalytic site. Before the reaction starts, the catalytic ions are caged by DMNP-EDTA and cannot be used for TdT, as the affinity towards DMNP-EDTA is greater than that towards TDT. Then, exposure to UV light breaks the structure of the caging molecule and triggers cage release back to the reaction environment. As TdT uses the catalytic ion again to perform another round of nucleotide addition and the reaction is stopped by adding an excessive amount of ion-caging molecule. This form of regulation is not a reversible reaction, and the length of the homopolymer structure depends on the irradiance and time of UV light on the synthetic product.

#### 2.3.2 TdT with terminator-modified nucleotides

The competitive method is improper for synthesizing sequence-specific ssDNA without ultimate optimization because it cannot add nucleotides in order and inevitably generates a homopolymer sequence. Therefore, based on the chemical method, the use of 3′ modified nucleotides was proposed to prevent further extension after the addition of one nucleotide [45], [46]. Inspired by phosphoramidite methods, 3′-modified nucleotides with chemical blockers are known to be used as substrates for various DNA polymerases, which can be cleaved by chemical solutions. Therefore, it is expected that the cyclic addition of 3′ blocked nucleotides with TdT will be developed shortly. In addition to chemically cleavable nucleotides, TdT incorporates 3′‐O‐nitrobenzyl and 4,5‐dimethoxy‐2‐nitrobenzyl groups, which are prone to UV cleavage [47], [48]. It was proposed that TdT uses a nitrobenzyl-bound dNTP (NZ-dATP) to add one correct nucleotide onto the 750 bp blunt-end dsDNA template. The addition of NZ-dATP did not increase the DNA length, and extension occurred only when dNTP was added after exposure to UV light. UV-A light successfully degrades the nitrobenzyl group without causing DNA damages. However, there is a controversy. As the number of oligonucleotides that accept NZ-dATPs as its substrate is so small, that even after blocking, there are some fractions of remaining unblocked nucleotides that extend their length after unmodified nucleotide extension [49] To enhance the incorporation of such reversible nucleotides, enzyme engineering and searching new chemical moiety are required.

#### 2.3.3 TdT reversely coupled to nucleotides with a linker

Using modified nucleotides requires enzyme engineering to incorporate bulky substrates and enlargement of the catalytic site, which may change the enzyme kinetics. This is due to the inherent characteristics of DNA polymerase, which has variable replication fidelity depending on the shape and size of the bases [50]. Although TdT has a wide variety of nucleotide preferences, 3′ OH blocked nucleotides have a poor incorporation rate compared to the naturally-occurring nucleotides with a free 3′ terminal hydroxyl group [51]. Therefore, although the method seems straightforward, finding and developing 3′ reversely blocked nucleotides with rapid enzyme kinetics is difficult. To solve this problem, a strategy was proposed for synthesizing a single, 3′ unblocked nucleotide, propargylamino-dNTPs, after forming a covalent bond with TdT [52]. The method requires modifying the base analog side of the nucleotide and synthesizing TdT using freely available hydroxyl groups. The covalent complex of TdT, tethered to a link with dNTP, inhibits further extension until it is cleaved by light at 365 nm and opens the 3′ end to continue the synthesis cycle. This has the side effect of leaving a propargylamino scar on the nucleobase, but the PCR amplification of the product successfully proceeds without any errors. In particular, this experiment has very little effect on enzyme kinetics because the 3′ end of the nucleotide remains the same as the natural nucleotide. The method succeeds in synthesizing 10mer oligonucleotide, yielding 93–98% per cycle.

## 3 Storage

The synthesized DNA is stored as dsDNA form for long-term stability. Since the durability of DNA is influenced by the medium they are stored in, the user should consider the appropriate method. Normally, DNA only requires dryness and ambient temperatures to be stored efficiently. Freezing DNA at a temperature between −20 and −80 °C, or in liquid nitrogen is sufficient, and dehydrated DNA can even be stored at room temperatures. But those methods inevitably encounter damages due to environmental factors like hydrolysis and UV irradiation [53]. Instead, DNA can be encapsulated in nanoparticles such as silica layers, iron oxide, and salts for higher stability under harsh conditions [6], [54], [55]. Cells can also be an *in vivo* storage medium by inserting DNA in bacterial plasmids, using genetic engineering systems [56], [57].

## 4 Reading: Selective retrieval of DNA for sequencing

During the encoding process, each DNA can be designed to have its own ‘barcode’ block, using error-correction codes. By doing this, a specific sequence can be read when required as in modern random-access memory [58]. When the desired data need to be decoded, the target DNA is selectively retrieved and amplified from the pool, using corresponding primers specific to the barcode block. In this step, selective amplification by polymerase chain reaction (PCR), keeps data from being volatile and needs a DNA template for synthesizing new strands. Then, the amplified DNA is sequenced for decoding. Most of the sequencing methods used are sequencing by synthesis (SBS) or nanopore sequencing. Since both of the methods requires ssDNA to initiate ‘reading’, we would like to introduce some mechanisms which generate amplified ssDNA from dsDNA. Although all the examples are given lack actual application in data reading currently, they are expected to be useful methods for obtaining the target DNA nevertheless (Table 2).

**Table 2:** Efficiencies of enzymatic ssDNA amplification.

Production method	Enzymes	Template	Length (nt)	Yield^a^	Application	Ref
aPCR	DNA Polymerase	ssDNA plasmid, lambda phage dsDNA	15,000	695 ± 35 ng/kb	Aptamer generation	[65]
		ssDNA plasmid, dsDNA fragments	449–3356		DNA origami scaffold synthesis	[64]
RCA	Phi 29 polymerase	Circular DNA	378		ssDNA synthesis	[81]
SDA	DNA polymerase, nicking endonuclease	ssDNA	500–1000	99.71%/kb	Synthesis of codon-usage variants of lacZα and 74 challenging Drosophila protein antigens	[110]
	Single stranded binding protein, DNA polymerase, nicking endonuclease	dsDNA (PCR product)	500	10-fold	ssDNA synthesis	[86]
			1000	10-fold		
			5000	12-fold		
SNAPCAR	DNA Polymerase	acrydite-labelled dsDNA (PCR product)	1650	50–70%	DNA origami scaffold synthesis	[96]
MeRPY	DNA Polymerase	acrydite-labelled dsDNA with uracil (PCR product)	89–3115	greater than70%	DNA origami scaffold synthesis	[99]

a Yields of ssDNA are converted from the ng/ul or mg/L from original article.

### 4.1 Asymmetric PCR

**Fig. 3:**
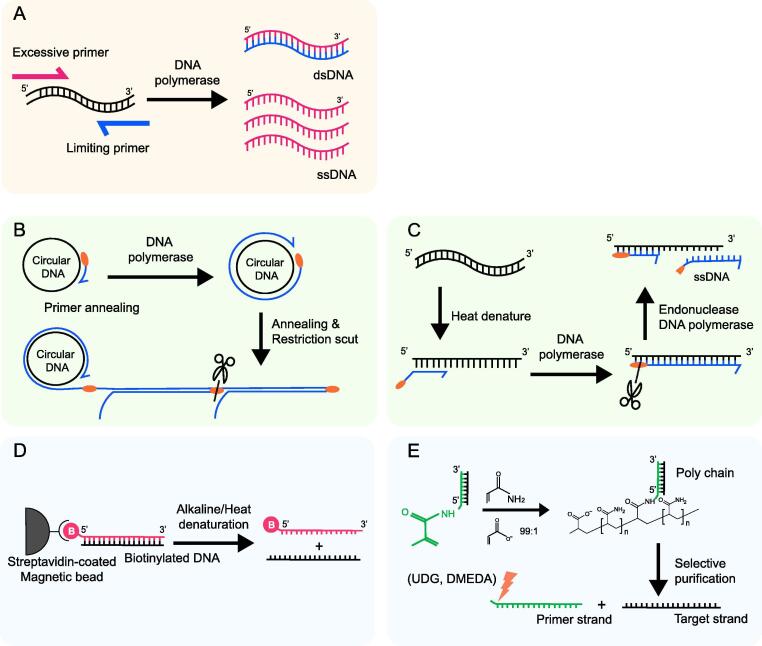
**Schematic illustration of enzymatic ssDNA amplification system**. (A) asymmetric PCR; Using unbalanced ratio of forward and reverse primers, an excessive amount of primer allows amplification of its target strand. (B) RCA; A highly processive DNA polymerase adds nucleotides continuously to the template (circular DNA). As the polymerase goes back to origin, the existing ssDNA unwinds itself to allow the polymerase synthesize another round of ssDNA. (C) SDA; DNA nicking site is generated by a set of primer and targeted for endonuclease activity. To the 3′ end of the nicking site, DNA polymerase extends the strand and displaces the former strand. (D) Biotin-streptavidin magnetic bead method; One of the strands is biotinylated and fixed onto magnetic bead. The unbound strand is rinsed off during denaturation process. (E) SNAPCAR/MeRPy-PCR; copolymerization of dsDNA with acrylamide and sodium acrylate (99:1 ratio) forms a DNA-tagged polymer. Using SNAPCAR, the target strand is recovered by alkaline denaturation and methanol precipitation. MeRPy-PCR allows recovery of both of target and primer strand by selective precipitation, using uracil-DNA glycosylase (UDG) and dimethylethylenediamine (DMEDA).

Asymmetric PCR (aPCR) reaction starts with a dsDNA template amplification using a set of primers in asymmetric concentration; one is excessive, while the other is insufficient (Fig. 3**A**) [59]. If the insufficient primers are completely consumed, the remaining excess primers amplify the annealed target ssDNA [60]. Since DNA polymerase adds complementary nucleotides to the template strand, the aPCR method has the advantage of ssDNA synthesizing directly from the template. In addition, ssDNA can be synthesized as much as possible using primers. However, because of the low processivity of DNA polymerase used in the early stages, aPCR has been mainly used for aptamer generation [61], [62] and gene detection [63], which requires short, 20–100 nt ssDNA. To synthesize longer DNA, Taq DNA polymerase was used to produce 3.3 kb ssDNA [64]. Soon after, they succeeded in producing 15 kb ssDNA using high-processive LongAmp Taq polymerase [65]. The overall synthesis yielded 2 pmoles of the 1000 nt product per 50 µl of the reaction volume.

The process of aPCR has several limitations. First, it requires highly selective purification methods because it is highly prone to the generation of dsDNA by-products [66], [67]. However, this can be partially overcome by the use of 3′ phosphate-blocked limiting primers, which decrease the mispriming and polymerization of DNA by-products [68]. Various purification methods, including gel extraction and immobilization on the column, have been suggested for this [61], [69]. Moreover, aPCR requires considerable optimization [70], [71]. The final yield of the desired ssDNA is highly dependent on the annealing temperature between the two primers [72], the number of amplification cycles [61], DMSO concentration [73], primer ratio, and the presence of a PCR enhancer such as L-ectoin [74]. Although it is sufficient to use a primer ratio between 1:50 to 1:100 with 30 cycles of PCR [59], the final efficiency varies over each condition. Thus, in order to achieve preferable conditions for long-length DNA synthesis, the durability of single-stranded DNA should be enhanced.

### 4.2 Isothermal amplification

PCR-based isothermal amplification aims to obtain large quantities of target sequences. This method is unique because thermal cycling is not required for the amplification step. In contrast to traditional PCR, isothermal amplification is carried out at a constant temperature under conditions such as water baths, cell surfaces, and living cell interiors that PCR cannot perform in [75].

Rolling circle amplification (RCA) is a method of rapidly synthesizing linear target sequences using highly processive polymerases that are using circular DNA as a template (Fig. 3**B**) [76], [77]. When the primer is annealed to the dsDNA template, DNA polymerase extends the primer by adding dNTPs and generates a linear ssDNA product at a constant temperature, ranging from 30 to 37 °C [77], [78]. As the polymerase returns to the origin of the template, the former strand is replaced by the strand displacement activity, creating a new template for continuous polymerization. The synthetic product is a tandem repeat of a complementary sequence of circular DNA [79]. Thus, the desired sequence can be obtained by including a restriction site in the primer and performing enzyme digestion after sufficient amplification is performed [80]. RCA was used for the synthesis of a 378 nt ssDNA, which is slightly longer than chemical synthesis (~200 nt) [81]. In terms of quantity, approximately 5 × 10^9^ copies of the 96 nt repeats were synthesized within 90 min [82].

Strand-displacement amplification (SDA) amplifies in isothermal conditions using a DNA polymerase activity with the *Hin*cII endonuclease (Fig. 3**C**) [83]. This method consists of two steps. First, a pair of primers amplifies the dsDNA template and generates the recognition site of *Hin*cII at the 5′ terminal end of the target sequence. This can be replaced by using a primer with a restriction site to a denatured ssDNA. Strand breaking occurs on the dsDNA containing the restriction site by *Hin*cII, and the DNA polymerase, such as a Klenow fragment, extends the primer strand. After the formation of a new strand, polymerization takes place at the restriction site of 3′ end again by nicking, allowing cyclic amplification [84], [85]. Based on this, synthesis of 0.5–5 kb ssDNA has been demonstrated, applying single-stranded binding protein-aided Sequenase 2.0 DNA polymerase and Nt.*Bsp*QI endonuclease. The final yield of the product was 68, 55, and 180 ng [86].

### 4.3 Separation of ssDNA from dsDNA

In addition to amplifying the desired strand from the template, data are obtained by denaturing dsDNA containing a specific strand. This can be done by selectively breaking down the unwanted strands or fixing the desired strands to the anchoring substance. Unlike PCR-based ssDNA synthesizing methods, an additional purification step is not required, because they can easily precipitate the desired strand and ensure high strand recovery yield.

The biotin-streptavidin magnetic bead method is derived from solid-phase DNA sequencing (Fig. 3**D**) [87]. To extract the desired DNA using this method, the data-encoding ssDNA was 5′-biotinylated and PCR amplified before storage, generating a complementary strand of the modified strand to make dsDNA. When the data needs to be decoded, the dsDNA was immobilized on magnetic beads and the remaining, non-biotinylated ssDNA that contains data can be purified by denaturing at 95 °C [88], [89], [90], [91]. The maximum length that can be synthesized using this method is 4808 nt [92], and the synthesis yield (recovery) decreases in proportion to the length [93]. Moreover, a fraction of the biotinylated strands still elutes, during the denaturation process. NaOH is involved in the hydrogen bond rupture between the biotinylated strand and streptavidin between the strands, causing reannealing between the strands and the loss of the desired product. Thus, the final yield of the reaction is highly dependent on synthesis length, NaOH concentration, and type of streptavidin-coated magnetic bead [94], [95].

Similar to the biotin–streptavidin-based strategy, selective nascent polymer catch-and-release (SNAPCAR) selectively anchors acrydite-modified dsDNA and denatures non-anchored, desired ssDNA (Fig. 3**E**) [96]. Using 5′-terminal acrylamide modified oligonucleotide as a primer, traditional PCR is performed to generate dsDNA. Then, co-polymerization of the PCR product with a 99:1 ratio of acrylamide and acrylate generates a dsDNA product linked to a poly (acrylamide-co-acrylate) chain [97], [98]. Following precipitation of the chain-bound dsDNA, incubation in alkaline denaturing conditions releases the mobile complementary strand. The immobile primer strand, that covalently attaches to the acrylamide chain, is pulled down thereafter. This method succeeded in purifying 1650, 3315, and 7301 nt ssDNA with 50 to 70% yield, which is the longest sequence length among the methods described in this review. In addition, the estimated cost of SNAPCAR is $0.35 per 1 nmol of ssDNA, which is about 10^3^ times cheaper than the streptavidin method. However, performing strand repair under standard laboratory conditions is inconvenient because the polymer must grow in low-oxygen conditions each time the PCR is run. Thus, methanol-responsive polymer PCR (MeRPy-PCR) was suggested, which compensates for the lack in the streptavidin method [99]. In MeRPy-PCR, deoxyuridine (dU) is site-specifically added to the acrydite-modified primer. After amplification, methanol purification, and denaturation of the mobile strand, the primer strand is recovered by uracil-DNA glycosylase (UDG) or dimethylethylenediamine (DMEDA). This method allows dual-purification of both the primer and target strand. The ssDNA synthesized by the above method showed more than 70% yield for the target strand and 20% for the primer strand, with a negative correlation between the length and the yield. The SNAPCAR and MeRPy methods can be applied to DNA scaffold origami construction. In addition, a DNA targeting strategy was proposed using ssDNA based on MeRPy-PCR [100].

## 5 Summary and outlook

In this mini-review, we discuss the current use of enzyme-based DNA synthesis and amplification methods for generating a digital data storage medium. Although most of the current mechanisms are based on chemical phosphoramidite synthesis, researchers today use enzymatic synthesis to achieve an efficiency rate similar to or even higher than the current techniques. This can generate a user-specific data-encoding strand by utilizing homopolymer generation or synthesizing designed sequences. Furthermore, enzymatic replication of DNA allows selective retrieval of the desired data. Although most enzymatic DNA synthesis methods still need to be developed with such limitations, TdT is thought to be the most promising method. Enzyme kinetics and innate properties of TdT provide a favorable starting point for applications in practical enzymatic DNA synthesis techniques.

Several challenges remain in reducing the final error rate. It is necessary to prevent ssDNA from forming a secondary structure when it exceeds a certain length. In this context, engineered TdT has been shown to withstand the high temperature of 47 °C, while preserving enzyme activity and inhibiting secondary structure [101]. In addition, the synthesis of high GC content is difficult due to the formation of hydrogen bonds between guanine repeats. This problem can also be partially solved with a lower catalytic ion concentration when synthesized with TdT [101]. Moreover, researchers today aim to apply enzymatic methods for generating larger assemblies, such as genome-scale sequences. Technologies such as Gibson assembly [65], [102], yeast assembly [103], and Golden Gate Assembly [104] have been extensively used in these research areas. As the maximum length of synthesizable DNA increases, we expect technological breakthroughs in the amount of data that could be stored in DNA. Overall, there is no doubt that DNA-based storage systems will become a highly efficient technology for replacing traditional storage mediums, and DNA-synthesizing enzymes will be the major factor for improving the process.

## CRediT authorship contribution statement

**Eojin Yoo:** Writing - original draft. **Donghui Choe:** Writing - original draft. **Jongoh Shin:** Writing - original draft. **Suhyung Cho:** Writing - original draft. **Byung-Kwan Cho:** Conceptualization, Supervision, Writing - original draft.

## Declaration of Competing Interest

The authors declare that they have no known competing financial interests or personal relationships that could have appeared to influence the work reported in this paper.
